# Pyrrhotite Fe_1−*x*_S microcubes as a new anode material in potassium-ion batteries

**DOI:** 10.1038/s41378-020-00188-0

**Published:** 2020-09-21

**Authors:** Yang Xu, Farzaneh Bahmani, Runzhe Wei

**Affiliations:** 1 Department of Chemistry, University College London, 20 Gordon Street, London, WC1H 0AJ UK; 2 National & Local United Engineering Laboratory for Power Batteries, Faculty of Chemistry, Northeast Normal University, Changchun, 130024 China

**Keywords:** Materials science, Chemistry

## Abstract

Potassium-ion batteries are an emerging energy storage technology that could be a promising alternative to lithium-ion batteries due to the abundance and low cost of potassium. Research on potassium-ion batteries has received considerable attention in recent years. With the progress that has been made, it is important yet challenging to discover electrode materials for potassium-ion batteries. Here, we report pyrrhotite Fe_1−*x*_S microcubes as a new anode material for this exciting energy storage technology. The anode delivers a reversible capacity of 418 mAh g^−1^ with an initial coulombic efficiency of ~70% at 50 mA g^−1^ and a great rate capability of 123 mAh g^−1^ at 6 A g^−1^ as well as good cyclability. Our analysis shows the structural stability of the anode after cycling and reveals surface-dominated K storage at high rates. These merits contribute to the obtained electrochemical performance. Our work may lead to a new class of anode materials based on sulfide chemistry for potassium storage and shed light on the development of new electrochemically active materials for ion storage in a wider range of energy applications.

## Energy storage: Developing electrodes for potassium-based batteries

Energy storage: Developing electrodes for potassium-based batteries. Microcubes of iron sulfide pyrrhotite constitute a promising electrode material for potassium-ion batteries. Potassium-ion (K^+^) batteries are being investigated as an alternative to lithium-ion batteries due to potassium’s abundance and low cost, but a challenge lies in discovering suitable materials for the battery’s electrodes. Yang Xu, and a team from University College London, United Kingdom, and Northeast Normal University, China, fabricated microcubes of pyrrhotite to use in K^+^ battery anodes to store ions. The anodes constructed by the team demonstrated a high capacity, with fast charge/discharge characteristics and stability that withstood repeated K^+^ insertion/extraction. Employing metal sulfides has cost and sustainability benefits, and the authors hope that their study can fuel further studies to realize the potential of K^+^ energy storage.

## Introduction

Research on new energy storage technologies has received great attention due to the inevitable depletion of fossil fuels and the need to reduce CO_2_ emissions to realize sustainable economic and societal growth. The last few years have seen rapidly increasing research activities on Na-ion and K-ion batteries (NIBs and KIBs) that use earth-abundant elements^1–5^. Materials are examined for their feasibility to serve NIBs and KIBs as alternatives to Li-ion batteries (LIBs), which could mitigate the potential supply risks and price increases of the current LIB industry. A recent theoretical study^6^, where an 18650 battery architecture was used and the weights of various battery components (cathode, anode, electrolyte, current collector, etc.) were taken into consideration, has suggested that KIBs have no considerable disadvantages over NIBs in terms of specific energy density. More importantly, there are critical benefits that favor the transition from NIBs to KIBs. First, the standard reduction potential of K is ~0.2 V lower than that of Na (−2.92 vs. −2.71 V), which could translate to a higher energy density. Second, unlike Na^+^, K^+^ intercalates reversibly into graphite^7^, which is a substantial attraction for industries to implement KIBs into the existing LIB manufacturing facilities. Third, K^+^ has a lower charge density compared to Na^+^, which could improve diffusion kinetics if intercalation sites and diffusion pathways are designed. These benefits highlight the commercial prospect of KIBs, and as a result, it is of utmost urgency to develop this exciting type of battery by exploring electrode materials that can store K^+^.

The major barrier to developing KIBs is the large size of K^+^ (K^+^/Na^+^/Li^+^: 152/116/90 pm) because it causes difficulties of K^+^ insertion and diffusion. An effective approach to overcome this barrier is to utilize crystal structures that have directional K^+^ diffusion pathways. This has resulted in a domination of intercalation-type materials for KIB cathodes, mainly including two classes of materials, i.e., layer-structured^8–10^ and tunnel-structured materials^11–14^, where K^+^ intercalation and diffusion are facilitated by the two-dimensional (2D) pathways in the former or three-dimensional (3D) pathways in the latter. In contrast, a variety of materials have been examined as KIB anodes owing to their various mechanisms to store K^+^ in the voltage window of anodes, which could alleviate the reliance on the directional diffusion pathways to some extent. For instance, disordered carbons perform well in storing K^+^ on their surfaces and realize high capacitive charge storage^15,16^. Metals and intermetallic compounds store K^+^ by an alloying mechanism and exhibit a high rate capability^17–21^. Conversion reactions can occur during K^+^ diffusion and often lead to a high K^+^ intake^22–25^.

Among the reported KIB anodes, metal sulfides have captured tremendous interest owing to their low cost and environmental friendliness^26^. In particular, 2D layered metal sulfides such as MoS_2_^27,28^, ReS_2_^29^, VS_2_^30^, and SnS_2_^31^ have been heavily studied. Their use as KIB anodes has been ascribed to a two-step K^+^ storage process that consists of an intercalation reaction and a subsequent conversion reaction (overlapping with an alloying reaction in the case of SnS_2_). The intercalation reaction uses crystallographic van der Waals gaps to accommodate K^+^, and the conversion reaction induces the reduction of the metal upon continuous K^+^ insertion. This kind of process generates a large capacity, being mainly contributed by the conversion reaction. Moreover, metal-sulfur bonds are less ionic than metal-oxide bonds, which is kinetically favorable for the conversion reaction, leading to good redox kinetics and reversibility^32,33^. These advantages have been the major motivations that drive the research of metal sulfides as KIB anodes to date, and some of them have indeed delivered high anode performance. It is worth noting that these sulfides have intrinsically low electronic conductivity, and as a result, the obtained performance often relies on the incorporation of carbonaceous materials that improve the electronic conductivity of the entire electrode matrix^29,31,34,35^.

Pyrrhotite, Fe_1−*x*_S (0 < *x* < 0.2), is one of the most important types of metal sulfides. This metal sulfide crystallizes in a distorted NiAs lattice with a superstructure of ordered iron vacancies, resulting in mixed Fe valences and nonstoichiometric formulas. The 3*d* electrons of Fe(II) and Fe(III) could overlap to increase the electron population within the conduction band^36,37^. Pyrrhotite exhibits a much higher electronic conductivity compared to its stoichiometric counterparts troilite (FeS) and pyrite (FeS_2_)^38,39^. Previous density functional theory (DFT) calculations have shown that the density of states (DOS) of pyrrhotite have no band gap near the Fermi level, indicating a metallic characteristic^40^. Owing to this characteristic, pyrrhotite has been used in a range of energy applications. Chen et al.^37^ reported pyrrhotite nanosheets as a highly efficient catalyst for oxygen evolution reactions due to the facilitated electron transfer process. Ahn et al.^41^ applied pyrrhotite as a functional additive in a sulfur cathode to improve its conductivity and trap lithium polysulfides in Li–S batteries. In the case of NIBs, pyrrhotite has served as not only a conductive core in heterostructured composites^42,43^ but also a single active material^44,45^. Surprisingly, there has not been any work on pyrrhotite in the application of KIBs. Bearing in mind that pyrrhotite, like other iron sulfides, undergoes a conversion reaction to store ions, it could be expected to deliver high KIB performance in combination with high electronic conductivity.

In this work, we report the first demonstration of pyrrhotite as an anode material for KIBs. Fe_1−*x*_S microcubes (MCs) were fabricated through a phase transition process, where pyrite FeS_2_ transformed to Fe_1−*x*_S during an annealing treatment at an inert atmosphere, and the cubical structure of FeS_2_ was preserved. Without the incorporation of carbonaceous materials, Fe_1−*x*_S MCs used as an anode exhibited a reversible capacity of ~420 mAh g^−1^ at 50 mA g^−1^ and great rate capability by retaining a capacity of over 120 mAh g^−1^ at a high current density of 6 A g^−1^. To the best of our knowledge, the obtained performance is the best among the reported iron sulfide anodes in the field of KIBs. Considering low cost and material sustainability, our work highlights the promise of iron sulfides in electrochemical ion storage.

## Results and discussion

### Structural and morphological analysis

Pyrrhotite Fe_1−*x*_S MCs were synthesized by direct, inert-atmospheric annealing of pyrite FeS_2_ MCs that were fabricated using a solvothermal reaction. During the thermal decomposition of FeS_2_, it was progressively transformed into Fe_1−*x*_S, and the crystalline grains became porous as sulfur gas escaped, which was reported in a previous investigation of the decomposition process and is described by the following equation^46^

$$\left( {1 - {\it{x}}} \right){\mathrm{FeS}}_{{\mathrm{2(}}{\it{s}}{\mathrm{)}}} \to {\mathrm{Fe}}_{1 - {\it{x}}}{\mathrm{S}}_{{\mathrm{(}}{\it{s}}{\mathrm{)}}}\left( {x = 0 - 0.2} \right) + 1/2\left( {1 - 2x} \right){\mathrm{S}}_{2(g)}$$

**Fig. 1:**
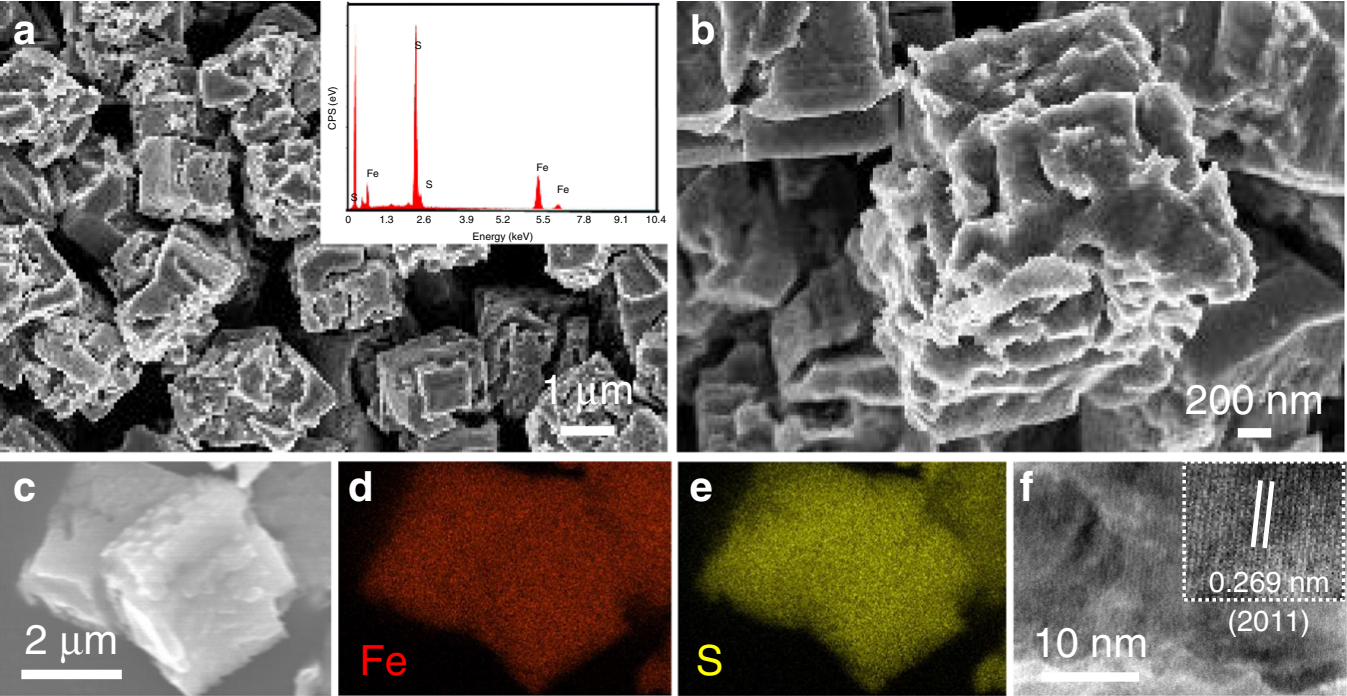
Structural characterization of Fe_1−*x*_S MCs. **a**, **b** SEM images, **c**–**e** elemental mapping, and **f** HRTEM image of Fe_1−*x*_S MCs. The inset in (**a**) is the EDS spectrum, and the inset in (**f**) is the lattice fringes

The scanning electron microscopy (SEM) image of FeS_2_ (Supplementary Fig. S1a) displays a cubical structure on a large scale with a small amount of polygonal structures. The high-magnification image (Supplementary Fig. S1b) shows that the MCs have a typical size of 1.5–2.5 µm and smooth surfaces. Energy-dispersive spectroscopy (EDS) results (inset in Supplementary Fig. S1a) demonstrate the existence of only Fe and S, and the S/Fe ratio is 2.23, being close to the formula FeS_2_ (characterized by X-ray diffractometry (XRD) and X-ray photoelectron spectroscopy (XPS) in Supplementary Figs. S3 and S4). The composition was further examined by elemental mapping (Supplementary Fig. S1c–e), where Fe and S were seen to be homogeneously distributed throughout the entire MC. Fe_1−*x*_S_2_ MCs successfully inherited the cubical structure on a large scale, as seen in the low-magnification SEM image (Supplementary Fig. S2). Close observations (Fig. 1a, b) reveal that Fe_1−x_S MCs have a similar size range as FeS_2_ MCs but rough surfaces and cracks that extend into the interior of the cubes. This is due to the escape of sulfur gas during thermal decomposition, as mentioned above. Such structural features could facilitate the penetration of the electrolyte and the diffusion of K^+^ into the MCs, indicating a good rate capability when Fe_1−*x*_S MCs are operated at high rates and only thin surface layers contribute to charge storage. EDS results (inset in Fig. 1a) reveal a S/Fe ratio of 1.11, suggesting a formula of Fe_9_S_10_, which is in accordance with the analysis of the pyrrhotite 5*C* structure^47^. The homogeneous distribution of Fe and S shown in the elemental mapping (Fig. 1c–e) once again confirms the phase purity of the MCs. The high-resolution transmission electron microscopy (HRTEM) image (Fig. 1f) shows a lattice spacing of 0.27 nm that corresponds to the spacing of the (2011) planes, which has been observed for pyrrhotite Fe_1−*x*_S in previous work^45^.

**Fig. 2:**
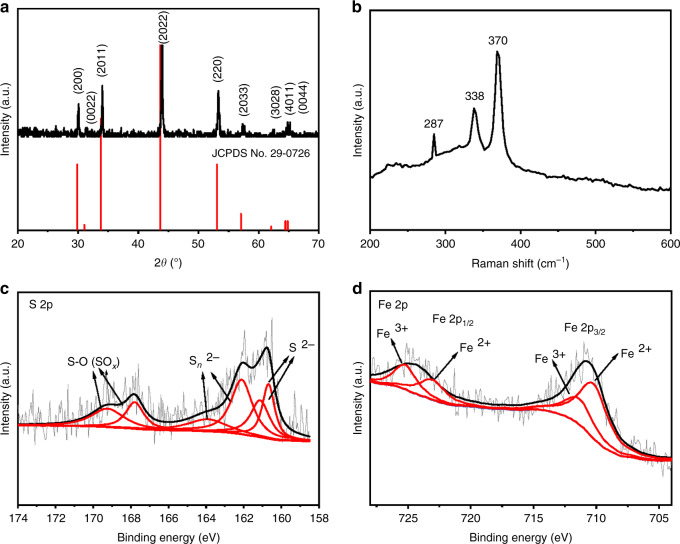
Phase characterization of Fe_1−*x*_S MCs. **a** XRD pattern, **b** Raman spectrum, **c** S 2p XPS spectrum, and **d** Fe 2p XPS spectrum of Fe_1−*x*_S MCs

Figure 2a and Supplementary Fig. S3 show the XRD patterns of the precursor and the annealed samples, respectively. All the peaks of the precursor can be well indexed to FeS_2_ with a cubic phase (JCPDS No. 42-1340). After annealing, all the peaks match well with pure hexagonal Fe_1−*x*_S (JCPDS No. 29-0726), suggesting the successful transformation from FeS_2_ to Fe_1−*x*_S. The Raman spectrum of Fe_1−*x*_S MCs (Fig. 2b) shows three visible bands at 287, 338, and 370 cm^−1^ that have been reported as characteristic bands of Fe_1−*x*_S^48,49^. XPS measurements were carried out to ascertain the chemical valences of the samples. In the survey spectrum of Fe_1−*x*_S MCs (Supplementary Fig. S4a), peaks belonging to Fe, S, C, and O can be observed, where the C and O peaks may originate from the adsorbed carbonaceous materials in the air^44,50^. The S 2p spectrum can be resolved into six peaks, as shown in Fig. 2c. The two peaks appearing at 160.8 and 161.6 eV can be assigned to 2p_3/2_ and 2p_1/2_ of S^2−^, respectively, while those at 162.1 and 164.1 eV are associated with 2p_3/2_ and 2p_1/2_ of S_n_^2−^, respectively^49,51^. In addition, the two peaks at 167.4 and 169.2 eV can be indexed to 2p_3/2_ and 2p_1/2_ of the S–O bond in the oxidized group (SO_*x*_), which has been reported from previous works^44,51^. The deconvoluted peaks of the Fe 2p spectrum (Fig. 2d) show the coexistence of Fe(II) and Fe(III), where the two peaks at 710.5 and 723.5 eV belong to Fe(II), and those at 713.3 and 726.1 eV belong to Fe(III)^52,53^. The XPS spectra of FeS_2_ (Supplementary Fig. S4b–d) have similar peak assignments of S_2_^2−^ and Fe^2+^ to those of previous literature^54,55^. It is noted that the N 1s peak appears in the survey spectrum of FeS_2_ but disappears in that of Fe_1−*x*_S. This is due to the presence of ethylenediamine (EDA) as a coordinating reagent in the growth of FeS_2_ and the elimination of EDA during thermal decomposition. We measured the Fourier transform infrared (FTIR) spectra of the samples (Supplementary Fig. S5), and the spectrum of FeS_2_ contains the bands of the characteristic vibrational modes of EDA, including the N–H stretching mode (3350 cm^−1^, overlapping with the O–H stretching mode), -NH_2_ bending mode (1630 cm^−1^), N–H bending mode (1400 cm^−1^) and C–N bending mode (1003 cm^−1^)^10,56,57^. These bands cannot be observed in the spectrum of Fe_1−*x*_S, which proves the elimination of EDA during the thermal decomposition of FeS_2_. EDA can coordinate with Fe^2+^ at the initial stage of the reaction to form a stable [Fe(amine)_2_]^2+^ complex^58^. The configuration of the complex confines the growth of FeS_2_ within one dimension (1D)^57^. Indeed, we observed 1D FeS_2_ nanowires that have low crystallinity after 8 h of the solvothermal reaction (Supplementary Fig. S6). However, the 1D structure gradually converted to a cubical structure when the reaction time was increased to 24 h, which was accompanied by a gradual increase in crystallinity. In addition, EDA can react with S to form a complex, octathioamine [NH_2_-(CH_2_)_2_-HN-S_8_^−^], that further degenerates to polythioamines [NH_2_-(CH_2_)_2_-HN-S_y_^−^] (*y* < 8)^58^, which prevents S from being fully reduced to S^2−^ and thus results in the formation of FeS_2_. This is different from a previous observation that Fe_1−*x*_S formed at the initial stage of a solvothermal reaction in which oleylamine was used to reduce S to S^59^. To summarize the structural and morphological analysis, Fe_1−*x*_S MCs were successfully fabricated through the decomposition of FeS_2_ MCs, where the cubical structure was inherited, and the surface of MCs became rough due to the release of sulfur.

### KIB anode performance

**Fig. 3:**
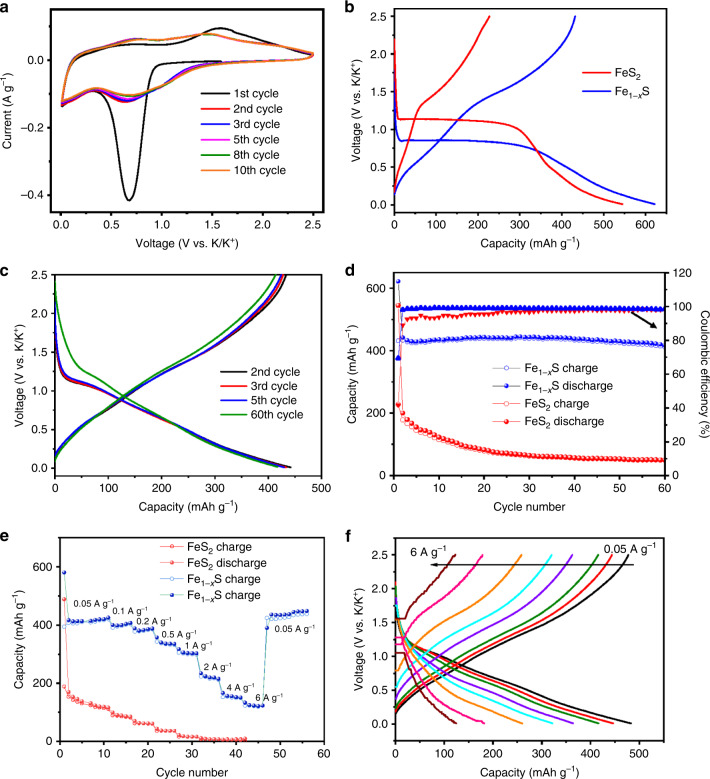
Electrochemical performance of Fe_1−*x*_S and FeS_2_ MCs. **a** CV curves of various cycles, **c** discharge/charge profiles at a current density of 50 mA g^−1^, and **f** discharge/charge profiles at various current densities of Fe_1−*x*_S MCs. **b** Discharge/charge profile of the first cycle, **d** cycling performance, and **e** rate capability of Fe_1−*x*_S and FeS_2_ MCs

The electrochemical performance of Fe_1−*x*_S MCs was evaluated as an anode material against metallic K in a coin-cell configuration and in a voltage range of 0.01–2.5 V (vs. K^+^/K), which is presented in comparison to the performance of FeS_2_ MCs. Figure 3a shows the CV curves of Fe_1−*x*_S MCs in the first 10 cycles at a scan rate of 0.01 mV s^−1^. The first cathodic scan was dominated by a strong peak centered at 0.7 V, which corresponds to the potassiation process that is dominated by the conversion reaction between K^+^ and Fe_1−*x*_S. The peak also includes the contribution from the formation of solid-electrolyte interphase (SEI), as its intensity significantly decreased in the subsequent cycles^45^. There are two broad peaks centered at 0.7 and 1.6 V in the first anodic scan, which are related to the multistep depotassiation process. From the second cycle onward, a broad peak ranging from 1.3 to 0.3 V can be seen in the cathodic scans, while the two anodic peaks shifted toward each other (0.8 and 1.4 V) in the anodic scans. There was no obvious loss of peak area in the successive cycles, signaling a considerable kinetic reversibility of K storage in Fe_1−*x*_S MCs after the initial (de)potassiation. The CV curves of FeS_2_ MCs (Supplementary Fig. S7) show a similar shape, but there is a significant peak current drop between the first and second cycles and a gradual reduction in the peak area, indicating the deteriorated reversibility of FeS_2_ MCs upon repeat K^+^ insertion/extraction. Figure 3b displays the discharge/charge profiles of the first cycle of both materials. The profiles exhibit one discharge plateau (0.8 V for Fe_1−*x*_S MCs and 1.1 V for FeS_2_ MCs) that corresponds to the strong peak in the first cathodic scan, and the less defined charge plateaus agree with the broad anodic peaks in Fig. 3a. The discharge plateau of Fe_1−*x*_S MCs is supposed to originate from the reaction between Fe_1−*x*_S and K^+^, with the formation of Fe, potassium sulfide and intermediate K-rich phases corresponding to the quantity of potassiation per Fe_1−*x*_S. A further potassiation step takes place when discharged to 0.01 V, which could result in the formation of not only Fe and K_2_S but also KFeS_2_, as seen in the reported work of K storage in iron sulfides^60,61^. The following charge process corresponds to a depotassiation reaction, for which it has been observed in both Na^62^ and K^61^ storage that instead of original iron sulfides, Na_*x*_FeS_*y*_ or K_*x*_FeS_*y*_ (possible multiphases) were the main products after the charge process; we speculate this phenomenon applies to our case, as no plateaus can be seen in the charge curve. From the second discharge/charge cycle onward, the transition between K_*x*_FeS_*y*_ and K_*x*+*x’*_FeS_*y*_ is responsible for the reversible K storage by inserting/extracting x’ mol K^+^, explaining the disappearance of the plateau in the 1st discharge process, which has been reported in iron sulfides for both Na^62,63^ and K storage^60,61,64^. The initial discharge/charge capacities of Fe_1-*x*_S MCs are 622/432 mAh g^−1^, giving rise to a high initial coulombic efficiency (CE) of 69%, which is much higher than that of FeS_2_ MCs (42%, 545/227 mAh g^−1^). The discharge/charge profiles of Fe_1−*x*_S MCs (Fig. 3c) overlap well with prolonged cycles, which agrees with the CV observations and shows good reversibility. Indeed, Fe_1-*x*_S MCs delivered capacities of 442 and 418 mAh g^−1^ in the 2nd and 60th cycles (Fig. 3d), respectively, resulting in a 95% capacity retention and a very small decay rate of 0.08% per cycle. The CE rapidly increased to 98% at only two cycles and remained above 98.5% in the following cycles. In contrast, the capacity of FeS_2_ MCs quickly dropped to 147 mAh g^−1^ after 6 cycles and remained only <50 mAh g^−1^ at the end of the cycles. Furthermore, Fe_1−*x*_S MCs also exhibited a great rate capability. As shown in Fig. 3e, the MCs delivered capacities of 413, 400, 383, 336, 303, and 220 mAh g^−1^ at current densities of 0.05, 0.1, 0.2, 0.5, 1, and 2 A g^−1^, respectively. Even at current densities as high as 4 and 6 A g^−1^, they retained capacities of 152 and 123 mAh g^−1^, respectively. Stable discharge/charge profiles can be seen at all testing current densities (Fig. 3f). After the current density was reduced to 0.05 A g^−1^, the capacity fully recovered to 437 mAh g^−1^, suggesting the electrochemical resilience of Fe_1−*x*_S MCs derived from the good conductivity and structural tolerance of the volume change during (de)potassiation. As expected, FeS_2_ MCs exhibited lower capacities compared to Fe_1−*x*_S MCs and failed to deliver meaningful capacities at current densities higher than 2 A g^−1^. To the best of our knowledge, the electrochemical performance of pyrrhotite Fe_1−*x*_S MCs is the best result among the performances of reported iron sulfide anodes in the field of KIBs^60,61^ and is even comparable to or better than those of some 2D layered metal sulfide-carbon composites^30,31,35,65^.

**Fig. 4:**
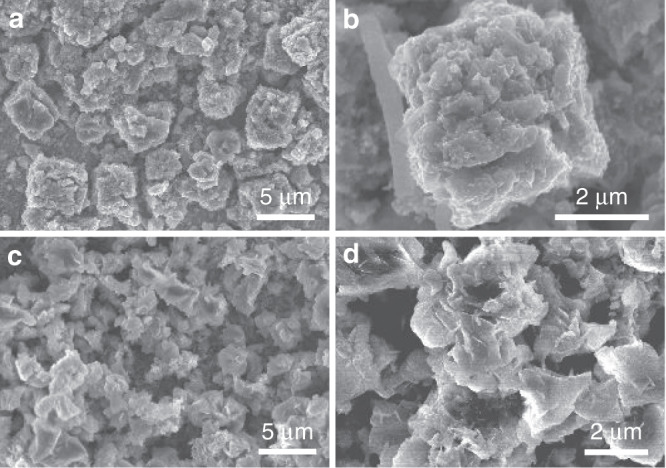
Postcycle characterization of Fe_1−*x*_S and FeS_2_ MCs. SEM images of Fe_1−*x*_S MCs (**a**, **b**) and FeS_2_ MCs (**c**, **d**) after cycling

The MCs were characterized after cycling, and the SEM images are shown in Fig. 4. The cubical structure of Fe_1−*x*_S was kept intact (Fig. 4a, b), despite the contour of the cubes being slightly less defined and the surfaces being rougher than those in the pristine state (Fig. 1a, b), which is due to the formation of SEI and volume change during the cycles. The preservation of the cubical structure indicates the stability of Fe_1−*x*_S during K storage even after undergoing a conversion reaction, which is responsible for the stable cycle life shown in Fig. 3d. However, the cubical structure of FeS_2_ could hardly be identified (Fig. 4c, d), and assemblies of nanosheets were observed instead. This indicates that the volume change causes the collapse of the cubes and that the microstructure is reconstructed after the conversion reaction. The reconstruction is governed by the 2D nature of the layer-structured FeS_2_^66,67^, resulting in the formation of nanosheets. It is worth noting that such reconstruction could introduce new electrode–electrolyte interfaces and thus increase the formation of SEI, which leads to a high charge transfer resistance (Fig. 6b). Future attention should be paid to encapsulating iron sulfides in carbonaceous materials or K^+^ conductive compounds in a way where space exists between the iron sulfide core and encapsulation shell to allow for volume changes, and the encapsulation shell serves as an artificial SEI to reduce the decomposition of electrolytes and the resultant charge transfer resistance across the solid–liquid interface. Nevertheless, the sharp contrast of the postcycle structures between the two anodes supports the superior electrochemical performance of Fe_1−*x*_S MCs over that of FeS_2_ MCs.

**Fig. 5:**
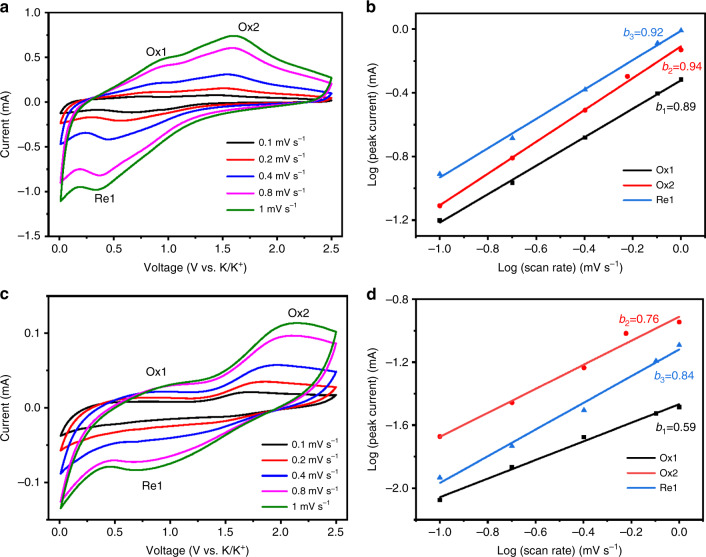
Kinetic study of Fe_1−*x*_S and FeS_2_ MCs. CV curves of Fe_1−*x*_S MCs (**a**) and FeS_2_ MCs (**c**) at various scan rates. The *b*-value determinations of Fe_1−*x*_S MCs (**b**) and FeS_2_ MCs (**d**)

**Fig. 6:**
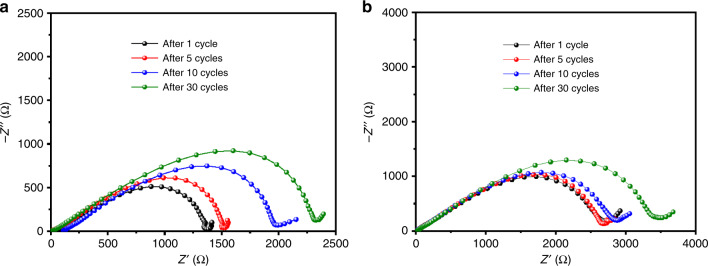
EIS measurements of Fe_1−*x*_S and FeS_2_ MCs. Nyquist plots of Fe_1−*x*_S MCs (**a**) and FeS_2_ MCs (**b**) after the 1st, 5th, 10th, and 30th cycles

### Kinetic study

A kinetic study was carried out to analyze the observed electrochemical behavior by using two electrochemical techniques. First, CV curves were recorded in a scan rate (*ν*) range of 0.1– 1 mV s^−1^. As shown in Fig. 5a–c, an increase in the peak current (*i*) and the separation of the reduction peak (Re, referring to potassiation) and oxidation peak (Ox, referring to depotassiation) were observed with increasing scan rate. Fe_1−*x*_S MCs exhibited a smaller Re-Ox separation and thus much less distorted curves than those of FeS_2_ MCs, indicating that a smaller polarization occurred in Fe_1−*x*_S upon fast K^+^ insertion/extraction, which agrees well with its better rate capability (Fig. 3e). According to the expression of *i* = *aν*^*b*^, where *a* and *b* are adjustable constants, the *b*-value can be extracted from the slope by plotting log(*i*) against log(*ν*), for which the value is 0.5 for a semi-infinite linear diffusion-controlled process and 1.0 for a surface-controlled process^68,69^. The *b*-value determinations of the two anodes are shown in Fig. 5b–d, where a better linear relationship was observed for Fe_1−*x*_S MCs (Fig. 5b) than for FeS_2_ MCs (Fig. 5d). The former has *b*-values of 0.92 (Re1), 0.89 (Ox1) and 0.94 (Ox2), which are higher than those of the latter (Re1: 0.84, Ox1: 0.59 and Ox2: 0.76). The comparison shows that more surface-dominated charge storage occurs in Fe_1−*x*_S MCs than in FeS_2_ MCs at high rates. This could be attributed to the rough surfaces and the cracks extending into the interior of the cubes (Fig. 1a, b), and the occurrence of surface charge storage could be maintained by the stability of the cubical structure, as shown in the last section. Both factors contribute to the high rate capability of Fe_1−*x*_S MCs.

Electrochemical impedance spectroscopy (EIS) was used as the second technique to analyze the charge transfer kinetics of the two anodes. Figure 6 shows the Nyquist plots recorded after the 1st, 5th, 10th, and 30th cycles. All the plots consist of a quasi-semicircle at high-to-medium frequencies and a small tail at low frequencies, where the former arises from charge transfer through electrode–electrolyte interface and the latter is ascribed to charge transport in the electrode^70,71^. Note that the diameter of the quasi-semicircles could qualitatively indicate the charge transfer resistance at the interface^71,72^. Fe_1−*x*_S MCs showed a much smaller diameter of the quasi-semicircle than that of the FeS_2_ MCs after the 1st cycle, suggesting a much smaller charge transfer resistance after the initial potassiation and the formation of SEI. The diameters of the quasi-semicircles increased during cycles for both anodes, but Fe_1−*x*_S MCs showed smaller diameters and thus smaller charge transfer resistance at all tested cycles. The Warburg coefficient is an indicator of ion diffusion behavior in solid electrodes and can be determined from the slope by plotting *Z’* against *ω*^*−*1/2^, where *Z’* is the real part of impedance and *ω* is angular frequency, for the low-frequency range (linear region of Nyquist plot)^73^. The plots (Supplementary Fig. S8) show a smaller slope for Fe_1−*x*_S MCs than for FeS_2_ MCs after every tested cycle, which suggests that the former has better K^+^ diffusion kinetics^73,74^. Therefore, the EIS results support the better cyclability of Fe_1−*x*_S MCs and agree with the observed stable structure.

### Conclusions

In summary, we reported a facile and scalable process to fabricate pyrrhotite Fe_1−*x*_S MCs through a phase transition from pyrite FeS_2_ while preserving the cubical structure. The MCs, with an estimated formula of Fe_9_S_10_, were tested as an anode in KIBs, and this is the first demonstration of pyrrhotite as an electrode material in the field. The anode exhibited a high reversible capacity of ~420 mAh g^−1^ and a good rate capability of ~120 mAh g^−1^ at 6 A g^−1^ as well as stable cyclability. Postcycle characterization demonstrated the high structural stability of Fe_1−*x*_S MCs upon repeat K^+^ insertion/extraction. Furthermore, a kinetic study revealed high surface-dominated charge storage that contributes to the obtained rate capability. We would like to use this work to highlight the promise of KIBs and hope to stimulate research on K storage, aiming to realize the practical use of KIBs in the future.

## Materials and methods

### Preparation of pyrite FeS_2_ and pyrrhotite Fe_1−*x*_S MCs

FeS_2_ MCs were synthesized via a solvothermal process. Typically, 1.5 mmol FeCl_2_·4H_2_O was dissolved in a mixture of deionized water and ethylene glycol (15 ml, 1:2 volume ratio) under stirring to form solution A. Sulfur powders (12 mmol) were dissolved in 10 ml ethylenediamine (EDA), and the suspension was stirred for 1 h to form homogenous solution B. Solution B was then slowly added to solution A and stirred for another 4 h. The as-prepared solution was transferred into a Teflon-lined stainless-steel autoclave and held at 200 °C for 24 h. The obtained FeS_2_ MCs were collected by centrifugation and rinsed with absolute ethanol and deionized water before drying in a vacuum at 80 °C. Fe_1−*x*_S MCs were obtained by annealing FeS_2_ MCs in an Ar atmosphere at 600 °C for 3 h with a heating rate of 5 °C min^−1^.

### Materials characterizations

Characterizations of the samples were performed by XRD (D4 ENDEAVOR, Cu Kα *λ* = 1.54 A), XPS (ESCALab 220i-XL), Raman spectroscopy (Renishaw Raman microscope spectrometer with a laser wavelength of 514 nm), Fourier transform infrared spectroscopy (Bruker platinum-ATR), transmission electron microscopy (JEOL JEM-2100), and SEM (JEOL JSM-7600F) equipped with an energy-dispersive X-ray spectrometer.

### Electrochemical measurements

Electrode films were prepared by coating a slurry made of 70% active material, 20% Super P and 10% sodium carboxymethyl cellulose (CMC) on a copper foil, which were dried at 110 °C under vacuum. The films were cut into disks as working electrodes with a mass loading of ~1.0 mg cm^−2^. Coin cells (R2032) were assembled in an Ar-filled glovebox with oxygen and moisture concentrations below 0.1 ppm. The cells contained the prepared working electrode, K metal as the counter electrode, a glass microfiber filter (Whatman, Grade GF/B) as the separator and 1 M potassium bis(fluorosulfonyl)imide (KFSI) in a mixture of ethylene carbonate/diethylene carbonate (EC:DEC = 1:1 volume ratio) as the electrolyte. Cyclic voltammetry (CV) and EIS measurements were performed on a VSP electrochemical workstation (Bio-Logic, France). Discharge–charge measurements were performed on a battery testing system (Land CT 2001A, China) at room temperature.

## Supplementary information

Supplementary information

## References

[1] Hwang J-Y, Myung S-T, Sun Y-K (2017). Sodium-ion batteries: present and future. Chem. Soc. Rev..

[2] Kim H (2018). Recent progress and perspective in electrode materials for K‐ion batteries. Adv. Energy Mater..

[3] Xu Y, Zhou M, Lei Y (2018). Organic materials for rechargeable sodium-ion batteries. Mater. Today.

[4] Yabuuchi N, Kubota K, Dahbi M, Komaba S (2014). Research development on sodium-ion batteries. Chem. Rev..

[5] Zhang W, Liu Y, Guo Z (2019). Approaching high-performance potassium-ion batteries via advanced design strategies and engineering. Sci. Adv..

[6] Eftekhari A (2018). On the theroretical capacity/energy of lithium batteries and their counterparts. ACS Sustain. Chem. Eng..

[7] Jian Z, Luo W, Ji X (2015). Carbon electrodes for K-ion batteries. J. Am. Chem. Soc..

[8] Hwang J-Y, Kim J, Yu T-Y, Myung S-T, Sun Y-K (2018). Development of P_3_-K_0.69_CrO_2_ as an ultra-high-performance cathode material for K-ion batteries. Energy Environ. Sci..

[9] Deng T (2018). Self-templated formation of P_2_-type K_0.6_CoO_2_ microspheres for high reversible potassium-ion batteries. Nano lett..

[10] Xu, Y. et al. Ammonium vanadium bronze as a potassium‐ion battery cathode with high rate capability and cyclability. *Small Methods* **3**, 1800349 (2018).

[11] Han J (2017). Investigation of K_3_V_2_(PO_4_)_3_/C nanocomposites as high-potential cathode materials for potassium-ion batteries. Chem. Commun..

[12] Recham N (2012). Preparation and characterization of a stable FeSO_4_F-based framework for alkali ion insertion electrodes. Chem. Mater..

[13] Zhang C (2017). Potassium Prussian blue nanoparticles: a low‐cost cathode material for potassium‐ion batteries. Adv. Funct. Mater..

[14] Chihara K, Katogi A, Kubota K, Komaba S (2017). KVPO_4_ F and KVOPO_4_ toward 4 volt-class potassium-ion batteries. Chem. Commun..

[15] Xu Y (2018). Highly nitrogen doped carbon nanofibers with superior rate capability and cyclability for potassium ion batteries. Nat. Commun..

[16] Yang J (2018). Enhanced capacity and rate capability of nitrogen/oxygen dual‐doped hard carbon in capacitive potassium‐ion storage. Adv. Mater..

[17] Zhang W, Mao J, Li S, Chen Z, Guo Z (2017). Phosphorus-based alloy materials for advanced potassium-ion battery anode. J. Am. Chem. Soc..

[18] An Y (2018). Micron-sized nanoporous antimony with tunable porosity for high-performance potassium-ion batteries. ACS Nano.

[19] Huang K (2018). Direct synthesis of 3D hierarchically porous carbon/Sn composites via in situ generated NaCl crystals as templates for potassium-ion batteries anode. J. Mater. Chem. A.

[20] Lei K (2018). A porous network of bismuth used as the anode material for high‐energy‐density potassium‐ion batteries. Angew. Chem. Int. Ed..

[21] Sultana I, Rahman MM, Ramireddy T, Chen Y, Glushenkov AM (2017). High capacity potassium-ion battery anodes based on black phosphorus. J. Mate. Chem. A.

[22] Sultana I (2017). K-ion and Na-ion storage performances of Co_3_O_4_–Fe_2_O_3_ nanoparticle-decorated super P carbon black prepared by a ball milling process. Nanoscale.

[23] Gao H (2017). CoS quantum dot nanoclusters for high‐energy potassium‐ion batteries. Adv. Funct. Mater..

[24] Li W (2019). Bismuth oxychloride nanoflake assemblies as a new anode for potassium ion batteries. Chem. Commun..

[25] Liu Y (2018). Boosting potassium-ion batteries by few-layered composite anodes prepared via solution-triggered one-step shear exfoliation. Nat. Commun..

[26] Tan H (2020). Metal chalcogenides: paving the way for high-performance sodium/potassium-ion batteries. Small.

[27] Dong Y (2019). Insights into the crystallinity of layer‐structured transition metal dichalcogenides on potassium ion battery performance: a case study of molybdenum disulfide. Small.

[28] Xu Y (2019). Enhancing potassium-ion battery performance by defect and interlayer engineering. Nanoscale Horiz..

[29] Mao M (2018). Flexible ReS_2_ nanosheets/N-doped carbon nanofibers-based paper as a universal anode for alkali (Li, Na, K) ion battery. Nano Energy.

[30] Zhou J (2017). Hierarchical VS_2_ nanosheet assemblies: a universal host material for the reversible storage of alkali metal ions. Adv. Mater..

[31] Fang L (2019). Few-layered tin sulfide nanosheets supported on reduced graphene oxide as a high-performance anode for potassium-ion batteries. Small.

[32] Sun X (2016). A high capacity thiospinel cathode for Mg batteries. Energy Environ. Sci..

[33] Ying X, Seon H, Yang K (2017). The application of metal sulfides in sodium ion batteries Adv. Energy Mater..

[34] Jia B (2018). Bamboo‐like hollow tubes with MoS_2_/N‐doped‐C interfaces boost potassium‐ion storage. Adv. Funct. Mater..

[35] Xie K (2017). Superior potassium ion storage via vertical MoS_2_ “nano‐rose” with expanded interlayers on graphene. Small.

[36] Wang H, Salveson I (2005). A review on the mineral chemistry of the non-stoichiometric iron sulphide, Fe_1-x_S (0≤ x≤ 0.125): polymorphs, phase relations and transitions, electronic and magnetic structures. Phase Transit..

[37] Chen S (2017). Highly active Fe sites in ultrathin pyrrhotite Fe_7_S_8_ nanosheets realizing efficient electrocatalytic oxygen evolution. ACS Cent. Sci..

[38] Shimada K (1997). Spin-integrated and-resolved photoemission study of iron chalcogenides. Phys. B.

[39] Pearce CI, Pattrick RA, Vaughan DJ (2006). Electrical and magnetic properties of sulfides. Rev. Mineral. Geochem..

[40] Honghong, F. et al. Pseudocapacitive sodium storage of Fe_1-x_S@N-doped carbon for low-temperature operation. *Sci. China Mater*. 10.1007/s40843-019-1220-2 (2019).

[41] Ahn JH, Veerasubramani GK, Lee S-M, You T-S, Kim D-W (2018). Improvement of Li-sulfur cell cycling performance by use of Fe_1-x_S@NC as a functional additive for chemical confinement of lithium polysulfides. J. Electrochem. Soc..

[42] Chen S (2019). Boosting sodium storage of Fe_1-x_S/MoS_2_ composite via heterointerface engineering. Nano-Micro Lett..

[43] Wan H (2018). Core-shell Fe_1-x_S@ Na_2. 9_PS_3.95_Se_0.05_ nanorods for room temperature all-solid-state sodium batteries with high energy density. ACS Nano.

[44] Xiao Y, Hwang J-Y, Sun Y-K (2017). Micro-intertexture carbon-free iron sulfides as advanced high tap density anodes for rechargeable batteries. ACS Appl. Mater. Interfaces.

[45] Li L (2017). Large-scale synthesis of highly uniform Fe_1-x_S nanostructures as a high-rate anode for sodium ion batteries. Nano Energy.

[46] Masset PJ, Guidotti RA (2008). Thermal activated (“thermal”) battery technology: Part IIIa: FeS_2_ cathode material. J. Power Sources.

[47] Elliot AD (2010). Structure of pyrrhotite 5*C* (Fe_9_S_10_). Acta Cryst..

[48] Weber, I., Böttger, U., Pavlov, S. G. & Hübers, H.-W. Raman investigation of iron sulfides under various environmental conditions. *46th**Lunar Planet. Sci. Conf.* (2015).

[49] Ma Q (2018). Iron-nitrogen-carbon species boosting fast conversion kinetics of Fe_1-x_S@C nanorods as high rate anodes for lithium ion batteries. Chem. Eng. J..

[50] Xiao Y, Hu C, Cao M (2014). High lithium storage capacity and rate capability achieved by mesoporous Co_3_O_4_ hierarchical nanobundles. J. Power Sources.

[51] Xiao Y, Hwang J-Y, Belharouak I, Sun Y-K (2017). Na storage capability investigation of a carbon nanotube-encapsulated Fe_1-x_S composite. ACS Energy Lett..

[52] Guo S-P, Li J-C, Xiao J-R, Xue H-G (2017). Fe_3_S_4_ nanoparticles wrapped in an rGO matrix for promising energy storage: outstanding cyclic and rate performance. ACS Appl. Mater. Interfaces.

[53] Li D (2018). Highly porous FeS/carbon fibers derived from Fe-carrageenan biomass: high-capacity and durable anodes for sodium-ion batteries. ACS Appl. Mater. Interfaces.

[54] Wen X, Wei X, Yang L, Shen PK (2015). Self-assembled FeS_2_ cubes anchored on reduced graphene oxide as an anode material for lithium ion batteries. J. Mater. Chem. A.

[55] Ma W, Liu X, Lei X, Yuan Z, Ding Y (2018). Micro/nano-structured FeS_2_ for high energy efficiency rechargeable Li-FeS_2_ battery. Chem. Eng. J..

[56] Steirer KX (2015). Nickel oxide interlayer films from nickel formate-ethylenediamine precursor: influence of annealing on thin film properties and photovoltaic device performance. J. Mater. Chem. A..

[57] Zhang F, Wang C, Huang G, Yin D, Wang L (2016). FeS_2_@ C nanowires derived from organic-inorganic hybrid nanowires for high-rate and long-life lithium-ion batteries. J. Power Sources.

[58] Zhu L, Richardson B, Tanumihardja J, Yu Q (2012). Controlling morphology and phase of pyrite FeS_2_ hierarchical particles via the combination of structure-direction and chelating agents. CrystEngComm.

[59] Liu W (2014). Solvothermal synthesis of pyrite FeS_2_ nanocubes and their superior high rate lithium storage properties. RSC Adv..

[60] Xie J (2018). Rational design of metal organic framework-derived FeS_2_ hollow nanocages@ reduced graphene oxide for K-ion storage. Nanoscale.

[61] Zhao Y (2018). High‐rate and ultralong cycle‐life potassium ion batteries enabled by in-situ engineering of yolk-shell FeS_2_@ C structure on graphene matrix. Adv. Energy Mater..

[62] Xiao Y (2017). Na storage capability invesitgation of a carbon nanotube-encapsulated Fe_1-x_S composite. ACS Energy Lett..

[63] Wang Y (2015). Uniform yolk-shell iron sulfide-carbon nanospheres for superior sodium-iron sulfide batteries. Nat. Commun..

[64] Chen C (2019). Graphene-encapsulated FeS2 in carbon fibers as high reversible anodes for Na^+^/K^+^ batteries in a wide temperature range. Small.

[65] Jia B (2018). Multirole organic-induced scalable synthesis of a mesoporous MoS_2_-monolayer/carbon composite for high-performance lithium and potassium storage. J. Mater. Chem. A..

[66] Wang D-Y (2015). Highly active and stable hybrid catalyst of cobalt-doped FeS_2_ nanosheets-carbon nanotubes for hydrogen evolution reaction. J. Am. Chem. Soc..

[67] Li Y (2018). FeS_2_/CoS_2_ interface nanosheets as efficient bifunctional electrocatalyst for overall water splitting. Small.

[68] Brezesinski T, Wang J, Tolbert SH, Dunn B (2010). Ordered mesoporous α-MoO_3_ with iso-oriented nanocrystalline walls for thin-film pseudocapacitors. Nat. Mater..

[69] Augustyn V (2013). High-rate electrochemical energy storage through Li^+^ intercalation pseudocapacitance. Nat. Mater..

[70] Aurbach D (2000). Review of selected electrode-solution interactions which determine the performance of Li and Li ion batteries. J. Power Sources.

[71] Xu Y (2013). Nanocrystalline anatase TiO_2_: a new anode material for rechargeable sodium ion batteries. Chem. Commun..

[72] Li Z (2015). High rate SnO_2_-graphene dual aerogel anodes and their kinetics of lithiation and sodiation. Nano Energy.

[73] Wang Y (2009). Synthesis and electrochemical performance of nano-sized Li_4_Ti_5_O_12_ with double surface modification of Ti(III) and carbon. J. Mater. Chem..

[74] Xu Y (2015). Enhancement of sodium ion battery performance enabled by oxygen vacancies. Angew. Chem. Int. Ed..

