# Supplementary material for: Pyrrhotite Fe1−xS microcubes as a new anode material in potassium-ion batteries
Source: Microsyst Nanoeng. 2020 Sep 21;6:75. doi: 10.1038/s41378-020-00188-0 (PMC8433425; doi:10.1038/s41378-020-00188-0)
Supplement: Supplementary file 1 — Supplementary information [file 41378_2020_188_MOESM1_ESM.docx]

**Supplementary Information for**

**Pyrrhotite Fe_1-x_S microcubes as a new anode material in potassium-ion batteries**

*Yang Xu,^1,*^Farzaneh Bahmani^2^ and Runzhe Wei^1^*

^1^ Department of Chemistry, University College London, 20 Gordon Street, London WC1H 0AJ, UK

^2^ National & Local United Engineering Laboratory for Power Batteries, Faculty of Chemistry, Northeast Normal University, Changchun 130024, China

* Corresponding author: Dr Yang Xu (email: y.xu.1@ucl.ac.uk; tel: +44 (0)20 7679 1502)


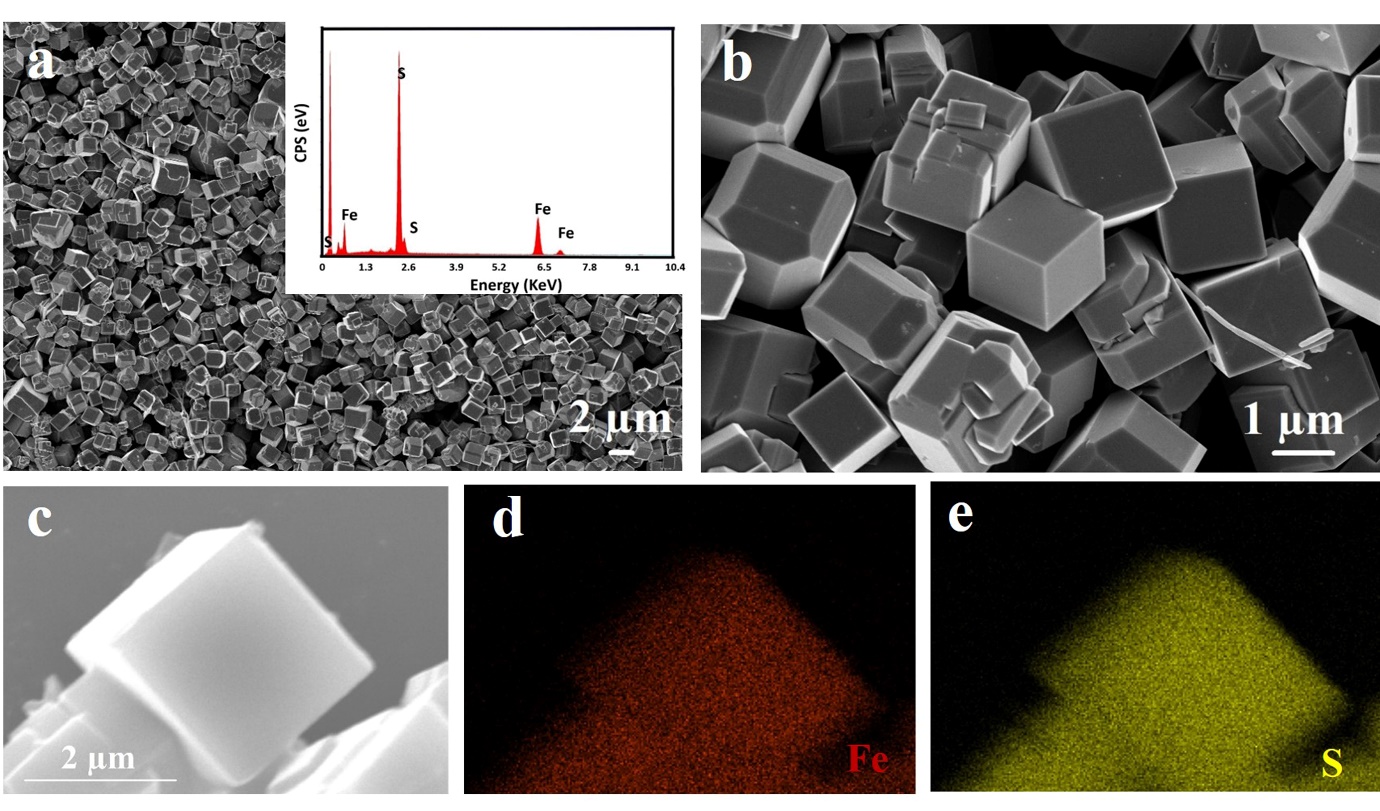


**Fig. S1** SEM images (a, b) and elemental mapping (c-e) of FeS_2_ MCs. Inset in (a) is EDS spectrum.


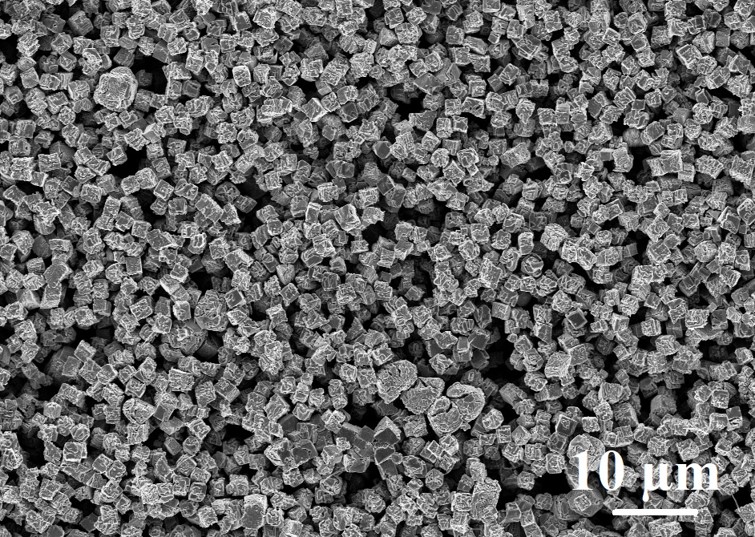


**Fig. S2** High-magnification SEM image of Fe_1-x_S MCs.


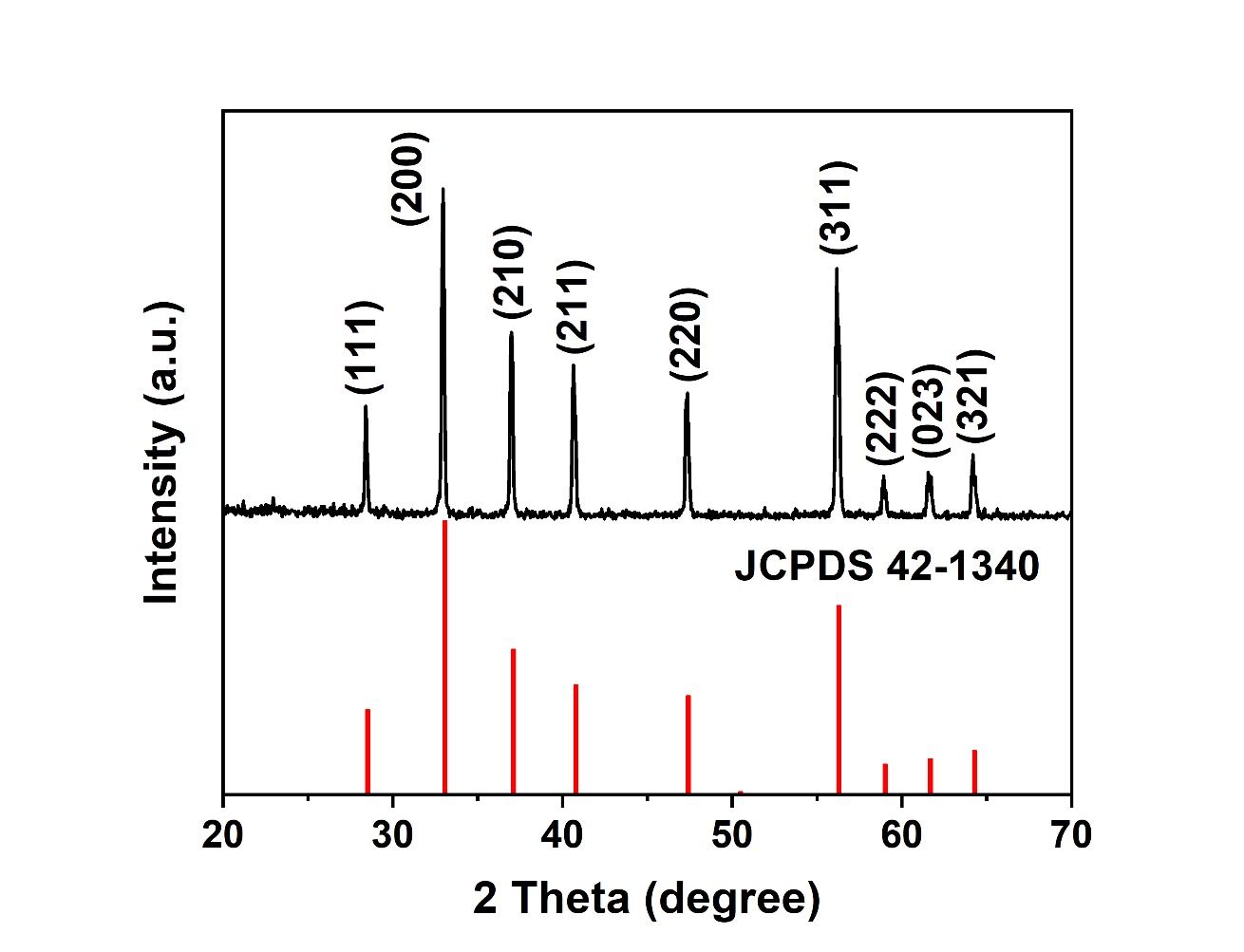


**Fig. S3** XRD pattern of FeS_2_ MCs.


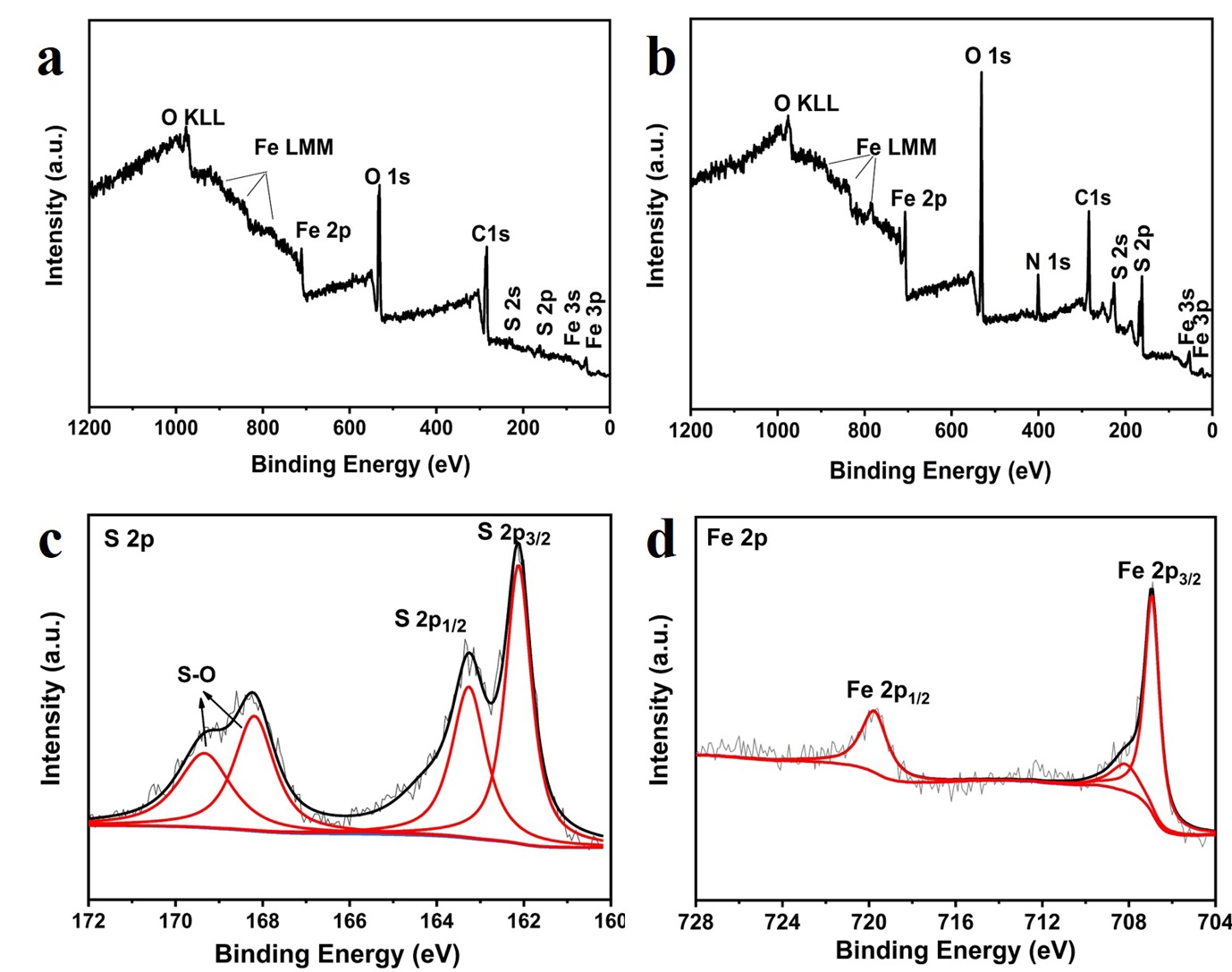


**Fig. S4** (a) XPS survey spectrum of Fe_1-x_S MCs. XPS survey spectrum (b), S 2p XPS spectrum (c), and Fe 2p XPS spectrum (d) of FeS_2_ MCs.


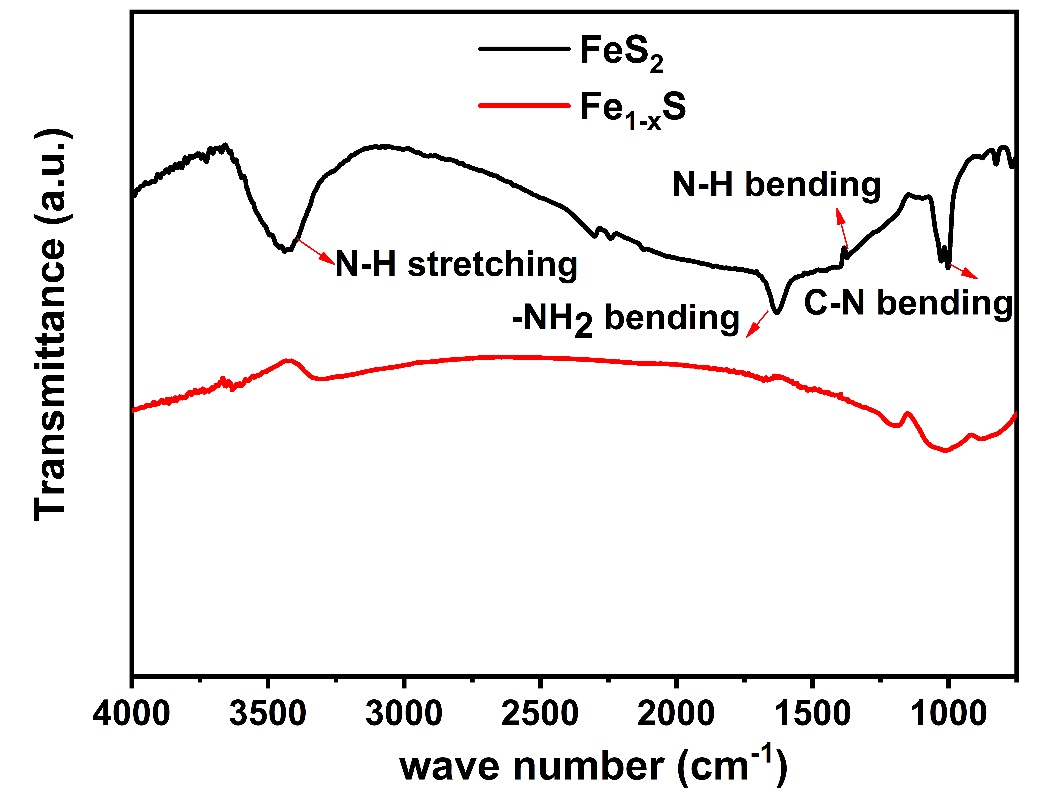


**Fig. S5** FTIR spectra of Fe_1-x_S and FeS_2_ MCs.


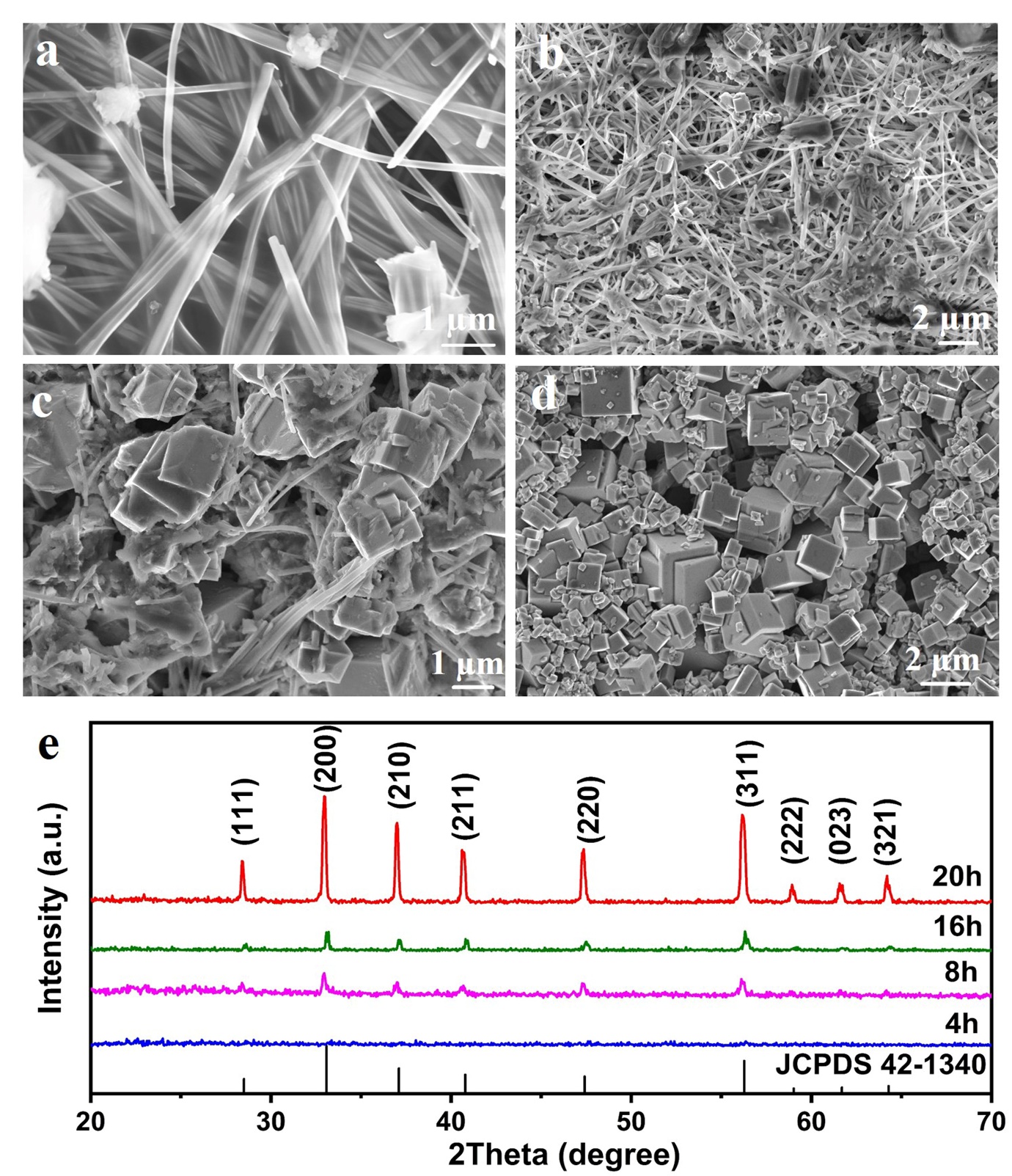


**Fig. S6** SEM images of intermediate FeS_2_ after 4 h (a), 8 h (b), 16 h (c) and 20 h (d) of solvothermal reaction. (e) XRD patterns of the corresponding intermediate samples.


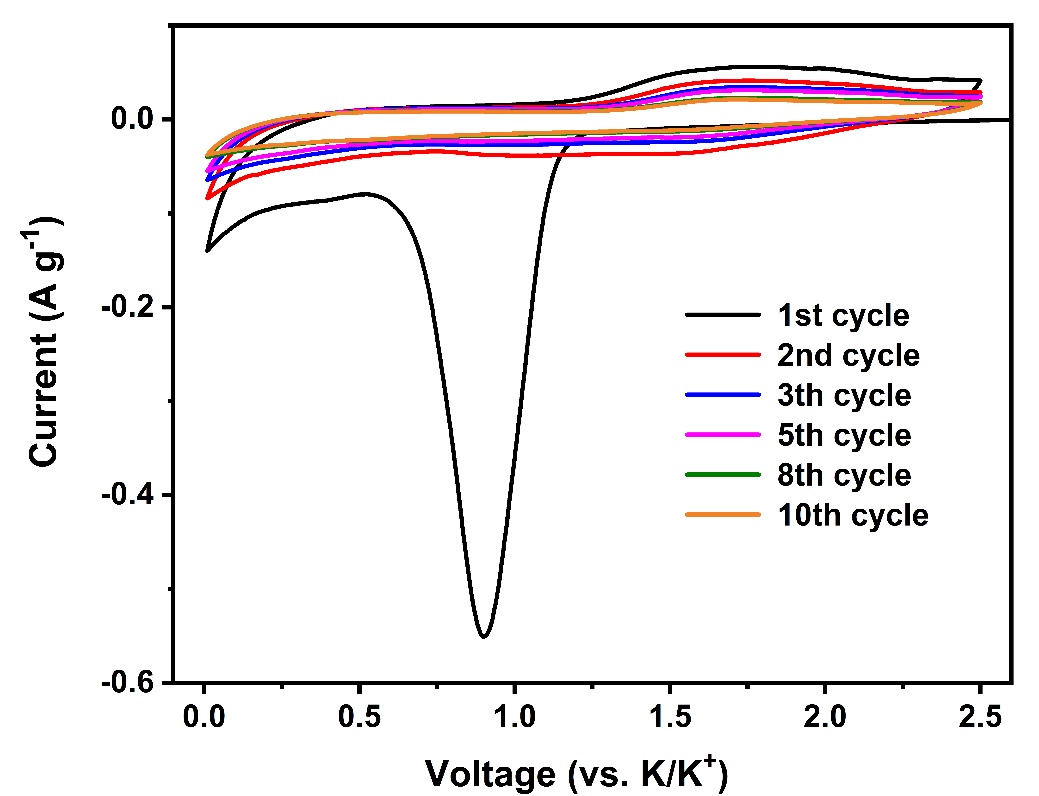


**Fig. S7** CV curves of various cycles of FeS_2_ MCs at the scan rate of 0.01 mV s^-1^.


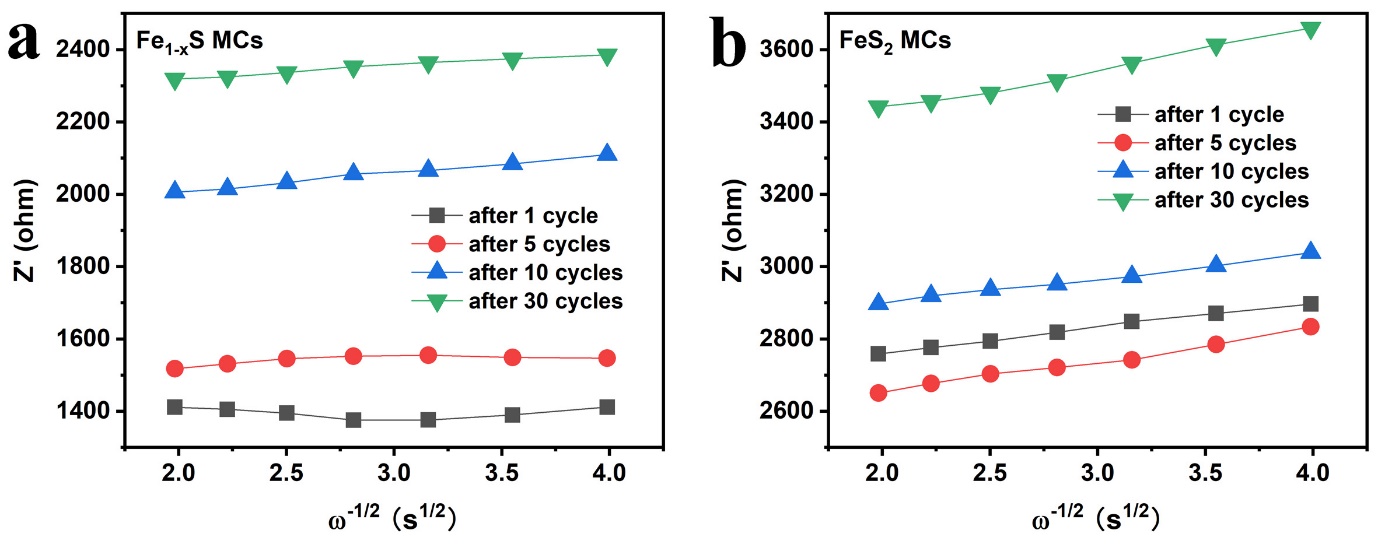


**Fig. S8** Plots of *Z’* vs. *ω^-1/2^* in the low frequency region obtained from EIS measurement for Fe_1-x_S MCs (a) and FeS_2_ MCs (b). Axes are set in same scales (*x*-axis: 1.75-4.25 s^1/2^ and *y*-axis: 1200 ohm) for a fair comparison of the slopes of the plots.
